# Development and Characterization of Gadolinium and Copper Reinforced Bioactive Glass: An In Vitro Study

**DOI:** 10.7759/cureus.55151

**Published:** 2024-02-28

**Authors:** Devika Bajpai, Arvina Rajasekar

**Affiliations:** 1 Periodontics, Saveetha Dental College and Hospitals, Saveetha Institute of Medical and Technical Sciences, Saveetha University, Chennai, IND

**Keywords:** biomaterial, bone regeneration, copper, gadolinium, alloplast

## Abstract

Introduction: Bioactive glass, an innovative alloplastic material utilizing a matrix of silica particles combined with calcium and phosphorus, has been widely employed for the regeneration of bony defects due to its bone-forming capabilities and biocompatibility. Nevertheless, it comes with several drawbacks, including a slow degradation rate, low mechanical strength, and susceptibility to fractures. To address these issues, the present research was done to develop and characterize a novel bioactive glass incorporating gadolinium (Gd) and copper (Cu).

Methods: The bioactive glass doped with Gd and Cu were synthesized and subjected to characterization through X-ray diffraction (XRD), scanning electron microscopy (SEM), and attenuated total reflectance-infrared (ATR-IR) analysis.

Results: The bioactive glass, enriched with Gd and Cu, underwent analysis using ATR-IR spectroscopy, XRD, and SEM. ATR-IR revealed characteristic silicate bands, while SEM indicated the presence of particles larger than 4 μm. XRD analysis identified the formation of Na_2_Ca_4_(PO_4_)_2_SiO_4 _(Silicorhenatite), Na_2_Ca_2_Si_3_O_9_ (Combeite), and wollastonite (calcium inosilicate mineral; CaSiO_3_). The crystalline nature of these compounds contributed to the favorable mechanical properties of the bioactive glass.

Conclusion: In summary, the creation of the innovative Gd-Cu-incorporated bioactive glass demonstrates favorable mechanical characteristics, suggesting significant promise for augmenting bone regeneration.

## Introduction

Periodontitis, characterized by inflammation impacting the supportive structures of teeth, including both soft and hard tissues, is primarily instigated by specific oral bacteria, leading to a host-mediated inflammatory response and subsequent loss of periodontal attachment [1,2]. Clinical interventions for periodontal disease can be either non-surgical, employing methods like combined scaling and root planing, or surgical, such as open flap debridement. When non-surgical approaches prove ineffective in resolving the issues, periodontal surgery may become necessary. Thus, surgical intervention, recommended only after non-surgical treatments have proven unsuccessful, typically involves addressing intrabony defects with various grafting materials that include autografts, allografts, xenogenic grafts, and alloplasts. Nowadays, alloplasts that are finely divided bioactive ceramics or hydroxyapatite, are known to stimulate the regrowth of alveolar bone [3-5].

In 1969, Larry Hench introduced bioactive glass, a pioneering material considered the earliest bioactive substance. This glass is recognized for its innovation in eliciting a positive action on osteoblasts [6]. Bioglass is theorized to be osteoinductive as it leads to the deposition of an apatite layer and has a strong bond with the host bone [7]. Although it has unique characteristics and extensive applications in bone tissue engineering, bioglass has limitations in terms of mechanical properties. Additionally, when compared to the Munson bone, it has an extremely porous nature [8].

Comprehensive research has been done on the integration of lanthanide elements into bioglass. Lanthanides have demonstrated the ability to mimic calcium functionally and address disorders related to bone density. Gadolinium (Gd), a member of the lanthanide group, has been extensively employed in various imaging techniques, such as magnetic resonance imaging (MRI) studies [9].

Similarly, the addition of copper (Cu) to the bioglass will improve the material's characteristics. This essential element has a crucial role in different metabolic activities, including soft tissue repair [10]. Copper ions play a vital role in promoting neovascularization during bone healing and act as antioxidants. Additionally, Cu ions significantly contribute to the regulation of gene expressions and the degradation of the extracellular matrix (ECM) [11]. Achieving comprehensive oral health recovery requires the development of new bioactive materials that interact with host tissues and enhance the surrounding environment. Therefore, this study aims to create and characterize bioactive glass reinforced with Gd and Cu.

## Materials and methods

Preparation of Gd and Cu incorporated bioactive glass

The bioactive glass, enriched with Gd and Cu, was produced by combining various chemicals (0.45 M tetraethyl orthosilicate, 10 ml of ethyl alcohol, distilled water each, and 2 ml of nitric acid). A gelatinous mixture was obtained after stirring all the constituents for 24 hours. Subsequently, other constituents (orthophosphoric acid, 0.6 moles; calcium nitrate, 0.245 moles; Gd nitrate, 0.05 moles; sodium hydroxide, 0.235 moles; and Cu nitrate, 0.05 moles) were introduced once the gel-like consistency was achieved. The blend was stirred at 100°C for three hours in a hot air oven and then poured into a muffle furnace at 700°C. Finally, the bioactive glass has been obtained after grinding the resulting mixture.

Morphological examination

The morphology of the Gd and Cu-incorporated bioglass network has been evaluated by utilizing a field-emission scanning electron microscope (FESEM) JSM-IT800 (JEOL Ltd, Tokyo Japan). Additionally, an energy-dispersive X-ray (EDX) analysis was done to identify biomaterials within Gd-Cu-bioglass. For an in-depth analysis of the chemical composition, Fourier-transform infrared (FTIR) spectra (Bruker Alpha II, Bruker, Billerica, MA, United States) were attained using attenuated total reflectance (ATR) sampling. Also, X-ray diffraction (XRD) analysis was conducted. The analysis utilized a copper K alpha (CuK) radiation source working at 40kV and 40mA, and the causing diffraction patterns spanning in the 2θ range, from 10 to 75 degrees, were noted [12].

## Results

Morphology and structure of Gd and Cu incorporated bioglass

**Figure 1:**
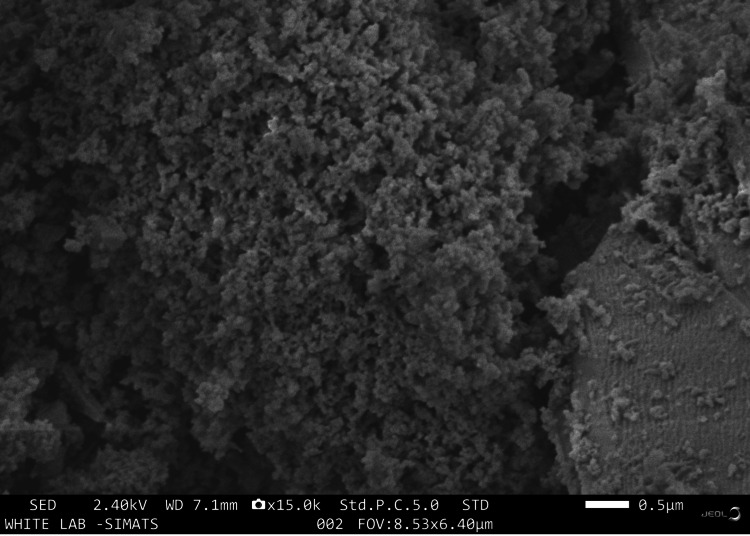
SEM analysis of the novel Gd-Cu bioactive glass Coarse particle size of more than 4 μm SEM: Scanning electron microscopy; Gd: Gadolinium; Cu: Copper

**Figure 2:**
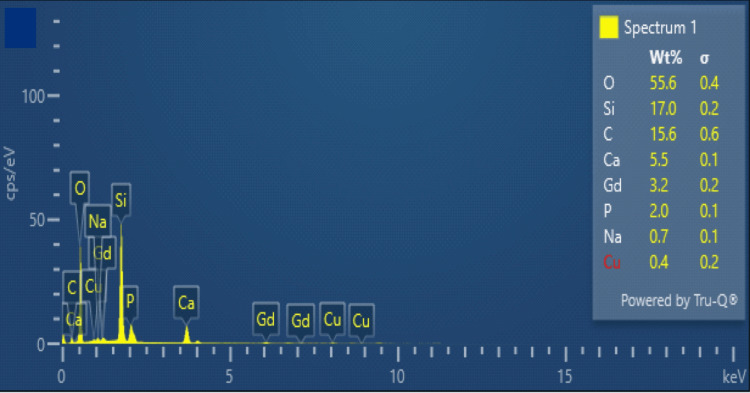
EDX quantitative analysis of elements in the novel Gd-Cu bioactive glass Sample composition EDX: Energy-dispersive X-ray; Gd: Gadolinium; Cu: Copper

The coarse particle size exceeding 4 μm was seen in SEM analysis (Figure 1). The spectra of EDX for the novel Gd-Cu-bioglass illustrated the sample composition, showcasing 3.2% Gd, 0.4% Cu, 17% silicon (Si), 5.5% calcium (Ca), and 2% phosphorus (P), all represented in the weight %, as shown in Figure 2.

**Figure 3:**
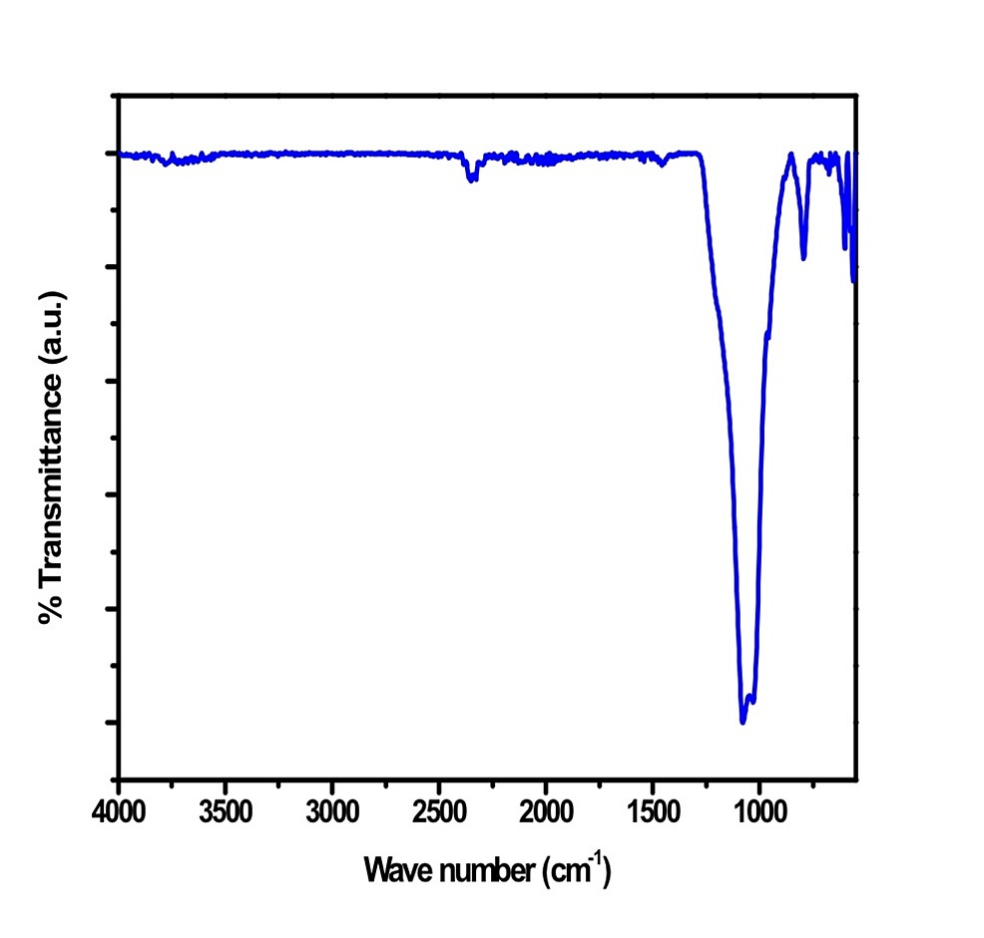
ATR-IR spectrum of Gd-Cu bioglass Vibration peaks were observed at various wave numbers ATR-IR: Attenuated total reflectance-infrared; Gd: Gadolinium; Cu: Copper

The attenuated total reflectance-infrared (ATR-IR) spectroscopic analysis of the Gd-Cu-bioglass network, as displayed in the given Figure 3, showed distinct silicate bands. These have been related to peaks of vibration at 1083 and 795 cm^-1^, indicative of the asymmetric along with symmetric stretching modes of Si-O-Si, correspondingly [13]. Furthermore, specific peaks were discerned in the spectra, including the observation of P-O-P stretching at 956 cm^-1^. Furthermore, the peak at 1043 cm^-1^ was associated with the bridging oxygen-related antisymmetric stretching modes of Si-O-Si tetrahedra. P-O bonds were identified as the source of a smaller peak at 598 cm^-1^, indicating a calcium phosphate P-O bending mode resembling that of crystalline apatite. Finally, a peak at 558 cm^-1 ^was related to the P-O vibrations symmetric stretching in the crystalline silicate.

**Figure 4:**
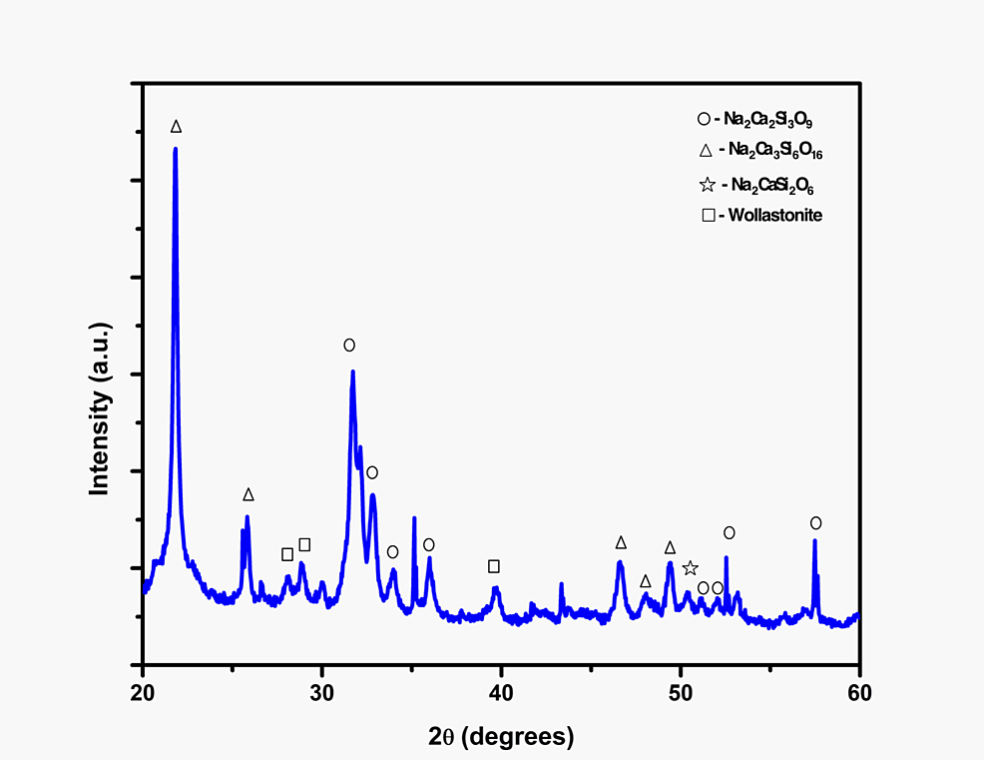
XRD analysis of Gd-Cu bioglass Diffraction patterns obtained in the 2θ range from 10 to 75 degrees XRD: X-ray diffraction; Gd: Gadolinium; Cu: Copper

The XRD examination, depicted in Figure 4, demonstrated a strong agreement with the standard PDF (Powder Diffraction File) #22.1455, confirming the crystalline phases development and displaying outstanding physical properties attributed to the densely packed finely crystalline powder of silica.

## Discussion

The primary objectives of periodontal therapy include halting the progression of the disease, preventing its recurrence, and restoring periodontal structures that have been compromised. The continuous quest for developing the biomaterial capable of regenerating bone defects has led to the discovery of these bioactive glass [14-15]. Bioglass has demonstrated osteostimulative properties in the context of bone regeneration [16]. A systematic review by Abushahba et al. affirms the effectiveness of bioglass in the treatment of osseous defects [17]. Nevertheless, this biomaterial is associated with several drawbacks, including a delayed resorption time and inferior mechanical properties.

This research involves creating a novel bioactive glass that incorporates Gd and Cu. This bioglass can be used both as a particulate graft and can be incorporated into a scaffold to enhance the outcomes of bone regeneration. The material's fundamental characteristic lies in its ability to gradually dissolve when exposed to water. After implantation, bioglass releases active ions, which will further lead to the deposition of the hydroxycarbonate apatite (HCA) [18].

Some research has observed angiogenic effects of bioactive glass, including secretion of vascular endothelial growth factor (VEGF) and augmented angiogenesis in vitro [19]. The combination of Gd and Cu will synergize both the biological and mechanical action of this material, which is also an objective of this study. The particle size of the novel bioglass exceeded 4 μm when viewed under SEM. The configuration obtained is noteworthy as it causes rapid adhesion, migration, and proliferation of proteins and cells and forms a new bone [20]. The similarity of the observed patterns in the ATR-IR spectra with that of hydroxyapatite suggests there was a conformational change in the structure that led to the transformation of bioglass powder (amorphous structure) to crystalline hydroxyapatite [21].

The incorporation of Gd into the silica network has a positive impact on the regulation of cellular activity and osteogenesis. Gd is naturally accumulated in human bones, with numerous studies demonstrating a notably significant amount of Gd precipitation on bone surface, suggesting bones as a preferential site for Gd accumulation [22]. Consequently, Gd-containing biomaterials have garnered significant interest globally [23]. Various in vitro studies indicate Gd enhances the growth of various cell types [24]. Research on magnesium alloys incorporating Gd and Zinc for orthopedic implants has shown that Gd possesses better strength and is resistant to corrosion [25]. Okada et al. demonstrated that Gd augments the deposition of calcium in mesenchymal stem cells (MSCs) [26]. However, the actual role of Gd-reinforced bioglass in osteogenesis is ascertained, and its contribution to controlling bone homeostasis is yet to be determined. Sandrine et al. emphasized the significance of Cu, asserting that it contributes to enhancing biological properties through controlled releases of its ions [27]. Incorporating copper in bioglasses serves multiple purposes. It is well-established that Cu exhibits improved antibacterial activity.

Limitations

The present study has limitations, including the absence of assessments on the resorption time, mechanical properties, cytotoxicity, and bone-forming potential of the composite. Consequently, future research should involve both animal and clinical trials to thoroughly evaluate the effectiveness of this Cu-Gd-incorporated bioactive glass in osseous regeneration.

## Conclusions

The successful fabrication of gadolinium-copper (Gd-Cu) bioactive glass involved the creation of hierarchical porous structures and the integration of the rare earth element Gd. At the concentrations employed, the introduction of Gd into the bioglass demonstrated favorable surface characteristics. Incorporating Gd and Cu into bioactive glass scaffolds represents a promising strategy to enhance their ability to address and heal bone defects.
